# Optimization of Oil and Tocopherol Extraction from Maqui (*Aristotelia chilensis* (Mol.) Stuntz) by Supercritical CO_2_ Procedure

**DOI:** 10.3390/antiox13070845

**Published:** 2024-07-15

**Authors:** Camila Sánchez, Alicia Rodríguez, Francisca Reinoso, Gretel Dovale-Rosabal, Nalda Romero, Alejandra Espinosa, María Elsa Pando, Benjamín Claria, Rodrigo Valenzuela, Cielo Char, Santiago P. Aubourg

**Affiliations:** 1 Department of Food Science and Chemical Technology, Faculty of Chemical and Pharmaceutical Sciences, University of Chile, Carlos Lorca Tobar 964, Santiago 8380494, Chile; camila.sanchez.s@ug.uchile.cl (C.S.); francisca.reinoso@ug.uchile.cl (F.R.); gretel.dovale@ug.uchile.cl (G.D.-R.); nromero@uchile.cl (N.R.); benjamin.claria@ug.uchile.cl (B.C.); cchar@ciq.uchile.cl (C.C.); 2 Department of Medical Technology, Faculty of Medicine, University of Chile, Independencia 1027, Santiago 8380000, Chile; ealejand@uchile.cl (A.E.); pandosanmartin@uchile.cl (M.E.P.); rvalenzuelab@uchile.cl (R.V.); 3 Department of Food Technology, Marine Research Institute (CSIC), Eduardo Cabello 6, 36208 Vigo, Spain

**Keywords:** freeze-dried maqui, supercritical fluid extraction, CO_2_, Response surface methodology (RSM) optimization, oil yield, tocopherols, validation, antioxidant capacity, total phenols

## Abstract

This study focused on the oil extraction from freeze-dried maqui (*Aristotelia chilensis*) by supercritical fluid extraction with carbon dioxide (SFE-CO_2_). The basic objective was to optimize the oil yield and the tocopherol concentration. A Box/Behnken experimental design was developed with three processing variables: supercritical pressure (74, 187, and 300 bar), temperature (35, 48, and 60 °C), and extracting time (30, 135, and 240 min). Multiple optimizations, based on the combination of factor levels at 274 bar, 240 min, and 60 °C, led to the highest oil yield and tocopherol values. The validation of the optimized conditions of maqui oil extraction led to an oil yield of 8% and values of 735, 53, and 97 (mg·kg^−1^ oil) for α-tocopherol, α-tocotrienol, and γ-tocopherol, respectively. A higher concentration of tocopherol compounds was observed when compared to the employment of the conventional extracting method. The optimized SFE-CO_2_ method led to an oil extract exhibiting higher Hydrophilic-Oxygen Radical Absorbance Capacity (H-ORAC) assay and total phenol content (22 μmol Trolox equivalents·g^−1^ oil and 28 mg gallic acid equivalents·g^−1^ oil) than the oil obtained by the conventional procedure. A practical and accurate oil extraction is proposed for obtaining tocopherol-enriched oil including high concentrations of valuable lipophilic antioxidants.

## 1. Introduction

Maqui (*Aristotelia chilensis* (Mol.) Stuntz) is a round small berry, 4 to 5 mm in diameter, with a dark violet to shiny black color [1]. This edible fruit, native to Chile and Argentina, is known for its high concentration of polyphenolic compounds, which are linked to numerous nutraceutical properties and health benefits, including antiatherogenic and cardioprotective properties, and prevention of oxidative stress, cognitive decline, and obesity [2,3,4]. Owing to its bioactive and physicochemical properties, maqui powder is a versatile product widely employed in food technology as a source of antioxidant compounds [5], for the preparation of preservative packaging [6], or as food tailoring focused on the preparation of diabetes- or gluten-free diets or the development of composite foods or nutraceuticals [7,8]. However, it is essential to consider that maqui fruit is harvested once per year, i.e., from December to February [3]. This seasonal availability creates the need to preserve the product and its beneficial properties over time [2]. Thus, different conventional and advanced technologies have been employed successfully to avoid the loss of its bioactive compounds [2,9].

Previous studies have addressed the extraction of bioactive compounds present in maqui. Such studies have concerned the antioxidant capacity of different kinds of extracts [10,11,12], the total polyphenol content [11,12], and the total anthocyanin content [12,13]. However, to the best of our knowledge, no previous research has been focused on the presence of tocopherol compounds in maqui.

Based on new challenges for the food industry, a wide range of eco-friendly methods are being used for lipid extraction from natural sources. Thus, ensilage [14], wet pressing [15], enzymatic hydrolysis [16], and urea concentration [17] can be mentioned. Among such methods, supercritical fluid extraction (SFE) methods have attracted great attention as reducing time and solvent usage while increasing yield and improving quality [18,19]. This method involves a combination of temperature and pressure, with carbon dioxide (CO_2_) being the most commonly used solvent due to its multiple advantages, i.e., non-toxic, non-flammable, cost-effective, and non-polluting. However, the primary advantage of using SFE with CO_2_ (SFE-CO_2_) lies in the quality of the extracted oil when compared to that obtained by conventional organic solvents [20]. However, there are still important concerns related to nutraceutical extraction methods that need resolution so that their tangible benefits are demonstrated before the industrial application of this innovative technology can be developed [21].

The present study focused on oil extraction from freeze-dried maqui by the SFE-CO_2_ extraction procedure. The basic objective was to optimize the oil yield and the concentration of the different tocopherol compounds (i.e., valuable lipophilic antioxidants) present in it. For it, an optimization process was carried out by a Box/Behnken experimental design following the response surface methodology (RSM) [22]. Antioxidant capacity and total phenol content of the oil extracted were also determined. A comparison to the conventional oil extraction (i.e., the Soxhlet procedure) regarding oil yield and tocopherol concentration was carried out.

## 2. Materials and Methods

### 2.1. Raw Material, Solvents, Chemicals, and Standards

The raw maqui substrate (*Aristotelia chilensis* [Mol] Stuntz) was purchased in the freeze-dried state from Lankorganic Spa (Lanco, Chile). Before starting the analysis, it was preserved at room temperature (ca. 20 °C) and protected from light and humidity in aluminum containers according to the manufacturer’s recommendations.

Methyl tricosanoate internal standard and GLC-463 reference standard for the gas/liquid chromatography analysis and indium standard were obtained from Nu-Chek-Prep, Inc. (Elysian, MN, USA). CO_2_, H_2_, and N_2_ gas cylinders were purchased from GasLab-Linde (Santiago, Chile).

α-, β-, γ-, and δ-tocopherol and α-tocotrienol standards, AAPH (2,2′-azobis (2-methylpropionamidine) dihydrochloride), fluorescein, Trolox (6-hydroxy-2,5,7,8-tetramethylchroman(E)-2 -carboxylic acid), gallic acid, and other chemical reagents were purchased from CalbioChem Merck (Santiago, Chile).

### 2.2. Extraction of Oil from Freeze-Dried Maqui Using SFE-CO_2_ and Soxhlet Procedures

SFE-CO_2_ extraction from the freeze-dried maqui was carried out in a Speed SFE system model 7071 supercritical CO_2_ equipment (Applied Separation, Allentown, PA, USA) using CO_2_ as solvent at 99.9% purity purchased from Gaslab-Linde (Santiago, Chile). The assembly of the column and its arrangement in the Speed SFE supercritical CO_2_ equipment for the extraction of maqui oil involved several steps. First, the extraction column was prepared and placed inside the oven. Then, using a pump, the pressure of the liquid CO_2_ was raised to the desired supercritical value and directed through the inlet valve to the extraction vessel, which was maintained at the desired temperature to operate in the required supercritical region. For the extraction process, the extraction tube was connected to the outlet valve and the ventilation valve, which was also connected to the flowmeter. Finally, the extraction valve was opened to obtain the oil for one hour at 5 mg·mL^−1^ CO_2_.

The conventional extraction of oil from freeze-dried maqui was carried out by employing the Soxhlet method in agreement with the American Oil Chemists’ Society (AOCS) Aa 4-38 procedure [23]. For this, 20 g of freeze-dried maqui were weighed, placed within a piece of filter paper, and enclosed in a second layer of filter paper to prevent material from escaping. The wrapped sample was then inserted into the extraction thimble, and 225 mL of petroleum ether was added to the extraction flask (previously weighed). The system was heated at a rate that allowed the solvent to drop from the condenser to the center of the wrapped sample at a rate of 150 drops·min^−1^. The extraction was conducted for 5 h, maintaining the solvent volume to compensate for losses due to evaporation. After the designated time, the system was cooled, and the extraction flask was disconnected for further processing. Petroleum ether was evaporated using a rotary evaporator, and to aid in the removal of the resulting residue, a stream of nitrogen was applied. The oil was then stored at −80 °C in amber glass containers, and nitrogen was introduced to displace oxygen in order to prevent oxidation until further use.

### 2.3. Experimental Design of RSM for the Oil Extraction by the SFE-CO_2_ Procedure

The Box/Behnken design based on the analysis of the RSM for the SFE-CO_2_ extraction of oil from the freeze-dried maqui was used. For it, three processing (i.e., independent) variables were considered, each of them at three different levels, i.e., supercritical pressure (74, 187, and 300 bar), temperature (35, 48, and 60 °C), and extracting time (30, 135, and 240 min). The independent variables were adjusted to maximize the responses of oil yield (%) and tocopherol content (mg·kg^−1^ oil) in the resulting oil.

The design included 15 experimental runs, three of them corresponding to the central points (187 bar, 48 °C, and 135 min) that allowed the experimental error to be estimated (Table 1). Furthermore, the experimental runs were conducted randomly to minimize the impact of hidden variables.

### 2.4. Optimization of the Process Variables of Oil Extraction to Maximize Response Variables

The optimization of the process variables was carried out using RSM according to the procedure of Myers and Montgomery [22]. The data obtained allowed us to build predictive quadratic polynomial models in terms of their regression coefficients for the processing variables. Furthermore, the combination of the dependent variables allowed for obtaining a theoretical value and establishing the predicted optimum of the yield (%) and tocopherol content (mg·kg^−1^ oil) values.

A mathematical model (Equation (1)) was obtained from RSM so that the effect of the independent variables could be predicted:

(1) $$Y=\beta_{0}+\sum_{i=1}^{k}\beta_{i}X_{i}+\sum_{i=1}^{k}\beta_{ii}X_{i}^{2}+\sum_{i=1}^{k}\sum_{j=1}^{k}\beta_{ij}X_{i}X_{j}+\varepsilon \;\;\;i<j\;$$

where *β_0_*, *β_i_*, *β_ii_*, and *β_ij_* represent the intercept, linear, quadratic, and interaction regression coefficients, respectively; *X_i_* and *X_j_* represent the independent variables; and *ε* corresponds to the random error. The regression coefficients were obtained by means of multiple regression analysis (*p* < 0.05).

An ANOVA of the regression parameters and the fitted model was performed (*p* < 0.05). The Statgraphics 19 statistical program (1982–2022 by Stat Point Technologies, Inc., Rockville, MD, USA) was employed.

### 2.5. Validation of the Optimized Formulation for Oil Yield and Tocopherol Compound Content

The optimized oil yield and tocopherol compound content, obtained from the optimized experimental design, were experimentally validated considering the theoretical or predictive optimized conditions of supercritical pressure, temperature, and extracting time. The physico-chemical properties of the oils extracted were comparatively analyzed according to the following quality indices.

The values of Commission Internationale de l’Éclairage (CIE-International Commission on Illumination) parameters L*, a*, and b* were evaluated in the maqui oils extracted by the SFE-CO_2_ and Soxhlet methods using a Lovibond Colorimeter (Amesbury, UK). Furthermore, the parameters chroma (C*_ab_) (Equation (2)) and hue angle (h_ab_) (Equation (3)) were calculated using the following equations:

(2) $${C^{*}}_{ab}={\left(a^{*2}+b^{*2}\right)}^{\frac{1}{2}}$$

(3) $$h_{ab}=\mathrm{arctg}\left(\frac{b^{*}}{a^{*}}\right)$$

where C*_ab_: chroma. h_ab_: hue angle. a*: red/green color. b*: yellow/blue color. 

The thermal analysis of the freeze-dried maqui and oil obtained from the optimized conditions and the conventional procedure was obtained by thermogram analysis with a PerkinElmer DSC 6000 equipment (Waltham, MA, USA). The thermograms were analyzed with the Pyris Player Software computer program, version 11.0.0.0449, where the temperatures (°C) of the beginning of the melting curve (T_Onset_), the maximum of the peaks (T_Peak_), and the end of the melting curve (T_Endset_) were obtained. Furthermore, the enthalpy of fusion (ΔH) was obtained from T_Onset_ and T_Endset_ of the sample (J·g^−1^). In the case of the freeze-dried maqui, the value of the glass transition temperature (°C) was also obtained.

Official AOCS procedures were employed for the lipid damage assessment [23]: free fatty acid (Ca 5a-40), peroxide (Cd 8b-90), and Total Oxidation (TOTOX) (Cg 3-91) values. The *p*-anisidine value (Cd 18-90), measured at 350 nm, and conjugated diene and triene formation at 233 nm and 268 nm, respectively (Ti 1a-64), were measured using a UV/Vis spectrophotometer, Unicam model UV3 (Cambridge, UK), with the Vision 32 software.

The antioxidant capacity was determined using the Hydrophilic-Oxygen Radical Absorbance Capacity (H-ORAC) assay according to the procedure described by Fuentes et al. [24]. First, the oil (30 g) was weighed into a 10 mL screw-cap tube, and 5 mL of a methanol/water (80/20) mixture was added. The mixture was vortexed for 30 s, sonicated for 15 min, and centrifuged at 1100× *g* for 25 min. The methanol/water extract was separated. A fluorescein (FL) stock solution was prepared, and a 0.075 M phosphate buffer (pH 7.4) was used for the preparation of Trolox standard solutions and samples. The reactions were carried out in a black 96-well plate. An aliquot (25 μL) of the phenolic extract diluted in methanol/water, or 25 μL of Trolox calibration solutions (12.5, 25, 50, and 100 μM), and 150 μL of fluorescein solution were added to each well. After tempering the plate (30 min at 37 °C), the reactions were initiated with the addition of 25 μL (150 mM) of AAPH (2,2′-azobis(2-amidino-propane) dihydrochloride). Readings were taken on an FLx800-TBID fluorescence reader (Biotek, Winooski, VT, USA), with excitation at 485 nm and emission at 528 nm measured from the top of the plate. The antioxidant capacity was expressed as μmol Trolox equivalent (TE)·g^−1^ oil.

The total phenolic content was determined spectrophotometrically using the Folin/Ciocalteu method as described by Fuentes et al. [24] using a Unicam UV/Vis spectrophotometer model UV3 (Cambridge, UK). The calibration curve was constructed using six different concentrations of the standard solutions of gallic acid (CalbioChem Merck, Santiago, Chile), from 50 to 500 μg·mL^−1^ (R^2^ = 0.9979). The results were expressed as μg gallic acid equivalents (μg GAE)·g^−1^ oil.

The composition of fatty acids (FAs) of the maqui oils extracted by SFE-CO_2_ and Soxhlet procedures was carried out by conversion to FA methyl esters (FAMEs) through the method described by the IUPAC [25]. The analysis of the resulting FAMEs was carried out according to AOCS Ce 1j-7 [26]. For it, a GLC Shimadzu gas chromatograph (Kyoto, Japan) equipped with a flame ionization detector, split injection system, and a capillary column (100 m × 0.25 mm i.d. × 0.2 µm) SP^TM^-2560 (Supelco, Bellefonte, PA, USA) was employed. The initial temperature of the oven was 160 °C and maintained for three minutes and then programmed with an increase of 1 °C·min^−1^ until reaching 230 °C. Both the injector and flame ionization detector temperatures were 240 °C. The carrier gas was hydrogen. For the qualitative determination, the retention times of the samples were compared to the retention times of a previously injected standard (GLC-463, Nu-Chek Prep, Elysian, MN, USA). The quantification of all the individual FAs (g·100 g^−1^ total FAs) was achieved using methyl tricosanoate (23:0 methyl ester) as an internal standard [26].

The presence of tocopherol compounds was determined by high-performance liquid chromatography (HPLC) according to the AOCS standard method Ce 8-89 [23] using an HPLC consisting of a Merck-Hitachi pump L-6200A (Merck, Darmstadt, Germany), a Rheodyne 7725i injector with 20 μL sample loop, a LiChro-CART Superspher Si 60 column (25 cm × 4 mm id, 5 μm particle size; Merck, Darmstadt, Germany), a Hitachi Chromaster 5440 fluorescence detector, and a PC with the Clarity chromatographic software, version 2.4.1.43, to process the chromatographic signal detected at excitation and emission wavelengths of 290 nm and 330 nm, respectively.

For the HPLC analysis of tocopherols, 80 µL of standard tocopherol solution and samples (3 µg·mL^−1^) were injected. The mobile phase consisted of propan-2-ol in hexane (0.5/99.5, *v*/*v*) flowing at a rate of 1 mL·min^−1^. For the qualitative and quantitative analyses, commercial standards of tocopherol compounds obtained from CalbioChem Merck (Santiago, Chile) were used. The results were expressed as mg tocopherols·kg^−1^ oil.

### 2.6. Statistical Analysis

The study was carried out in duplicate. The results are presented as average ± standard deviation (SD) and the analysis of variance (ANOVA) was performed. Differences between oils obtained from the Soxhlet or SFE-CO_2_ procedures were examined using the Tukey test and were considered significant at *p* < 0.05 (InfoStat program version 2020).

A goodness-of-fit test of the statistical analysis carried out in the study has been included as Supplementary Material (Table S1).

## 3. Results

### 3.1. Effect of Process Variables of SFE-CO_2_ Extraction of Maqui Oil: Analysis by RSM, Pareto Charts, and Regression Coefficients

The effect of the process variables on the oil yield (%) can be observed in Table 1. It is highlighted that when the extraction is carried out at low levels of supercritical pressure (74 bar), the yield is zero since no oil is extracted, while at high levels of supercritical pressure (300 bar), the oil yield increases significantly (*p* < 0.05). The highest yield obtained was 9.20%, corresponding to the highest pressure and extracting time and to a supercritical temperature of 48 °C.

**Figure 1:**
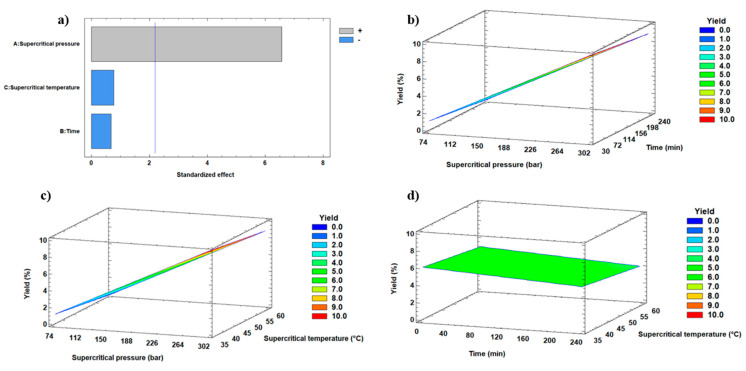
(**a**) Standardized Pareto diagram for oil yield (%) a higher value than the blue line mark indicates a significant effect, *p* < 0.05, (**b**) the RSM of pressure vs. time, (**c**) the RSM of pressure vs. temperature, and (**d**) the RSM of temperature vs. time. In (**b**–**d**) Figures, the third variable is held constant, and the surface height represents the value of the oil yield (%) extracted from the freeze-dried maqui using the SFE-CO_2_ procedure.

Figure 1 shows the effect of the three process variables on the oil yield (%) extraction through Pareto-type and response surface figures. The effect of the process variables that significantly influenced the yield was determined by statistical analysis using the RSM. The Pareto charts (Figure 1a) show the standardized effect and interaction for each response variable in decreasing order (i.e., a higher value than the blue line mark indicates a significant effect, *p* < 0.05); therefore, the pressure was the only variable having a significant (*p* < 0.05) positive effect on the oil yield. The response surface-type graphs show oil yields according to the value of the variables. In Figure 1b,c, a positive slope is observed so that whatever the extracting time and temperature were, an increased pressure led to yield increases.

**Figure 2:**
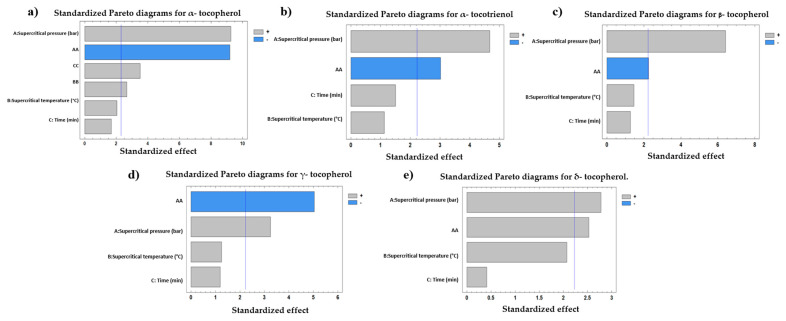
Standardized Pareto diagrams for (**a**) α-tocopherol, (**b**) α-tocotrienol, (**c**) β-tocopherol, (**d**) γ-tocopherol, and (**e**) δ-tocopherol. A higher value than the blue line mark indicates a significant effect, *p* < 0.05.

The effect of the independent variables on the tocopherol compound presence (mg·kg^−1^ oil) is shown in Table 2. Figure 2 shows that the supercritical pressure had a significant positive influence on the content of the five tocopherol compounds. Additionally, a negative effect was observed in the pressure-squared interaction for the α-tocopherol, α-tocotrienol, β-tocopherol, and γ-tocopherol values. Finally, the pressure/temperature interaction affected significantly and positively the δ-tocopherol content.

**Figure 3:**
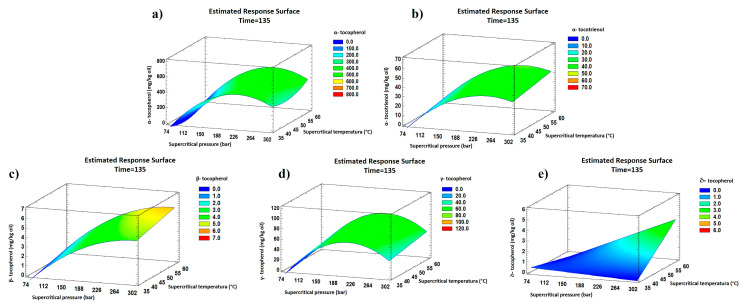
RSM figures of tocopherol compounds obtained from maqui oil by SFE-CO_2_ extraction. Effect of pressure vs. temperature on tocopherol compound values (mg·kg^−1^ oil): (**a**) α-tocopherol, (**b**) α-tocotrienol, (**c**) β-tocopherol, (**d**) γ-tocopherol, and (**e**) δ-tocopherol.

According to Figure 3, an increase in the supercritical pressure favored an increase in the tocopherol values. In the case of α-tocopherol, α-tocotrienol, β-tocopherol, and γ-tocopherol, a maximum value was obtained for pressure values of 226, 247, 286, and 212 bar, respectively, followed by a content decrease.

The lineal (Y_1_) and quadratic (Y_2–6_) polynomial equation adjusted for the predicted models of oil yield (Y_1_), and the contents of α-tocopherol (Y_2_), α-tocotrienol (Y_3_), β-tocopherol (Y_4_), γ-tocopherol (Y_5_), and δ-tocopherol (Y_6_) are described in Table 3. This table shows the regression coefficients of the predictive primer (Y_1_) and second (Y_2–6_) order polynomial model for the response variables. The results of fitting a multiple regression model describe the effect of the different process variables on the response variables considered for yield (%) and tocopherol concentration (mg·kg^−1^ oil) obtained from the freeze-dried maqui by the SFE-CO_2_ extraction.

### 3.2. Optimization of the Process Variables of SFE-CO_2_ Extraction of Maqui Oil

**Figure 4:**
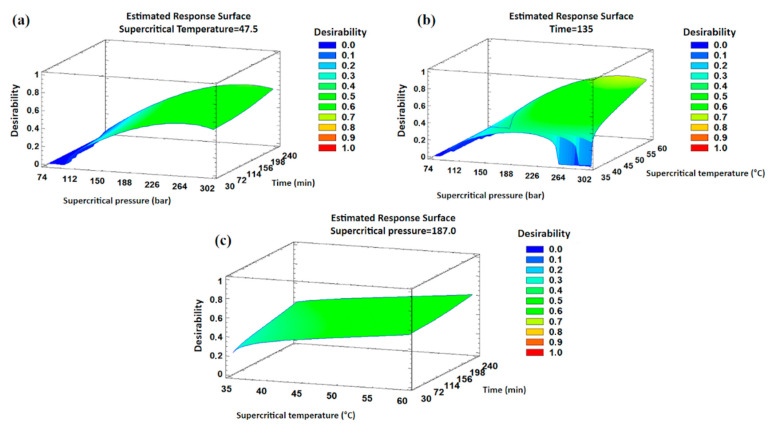
RSM graphs for the desirability value based on supercritical pressure, time, and temperature in maqui oil obtained from SFE-CO_2_ extraction. (**a**) Pressure vs. time, (**b**) pressure vs. temperature, and (**c**) temperature vs. time.

A study with multiple response optimization was conducted, aiming to maximize both the oil yield (%) and the tocopherol concentration (mg·kg^−1^ oil) simultaneously. Response surface graphs (Figure 4) were generated, illustrating the combination of factor levels where the “desirability” function is maximized within the specified region, which corresponds to higher values of pressure, time, and temperature.

Furthermore, the optimized combination showed a desirability value of 0.78 and was identified as corresponding to 274 bar, 60 °C, and 240 min, respectively. Regarding the oil yield, a 7.40% value would be expected for such a value combination. Furthermore, it would be expected to obtain the concentration values (mg·kg^−1^ oil) of 590 for α-tocopherol, 50 for α-tocotrienol, 6 for β-tocopherol, 71 for γ-tocopherol, and 3 (traces) for δ-tocopherol. 

### 3.3. Validation of the Optimized Formulation of SFE-CO_2_ Process Variables

The oil from the freeze-dried maqui was experimentally obtained using the SFE-CO_2_ extraction according to the variable values provided by the optimization process, i.e., 274 bar, 60 °C, and 240 min. The physico-chemical properties of such oil extract were determined and are presented now. A comparison to oil extracted by the conventional Soxhlet method is shown.

Regarding the oil yield (%), the optimized SFE-CO_2_ procedure led to a 7.65 ± 0.78^a^ value, which was found slightly lower (*p* < 0.05) than the one obtained by employing the conventional method (8.53 ± 0.16^b^).

Results on the color of the maqui oil extracted using the optimized SFE-CO_2_ and Soxhlet methods led to significant differences (*p* < 0.05) between the extraction methods for the L*, a*, and b* values. For the L* parameter, −10.68 ± 0.00^a^ and −12.35 ± 0.00^b^ values were obtained by using the optimized SFE-CO_2_ and Soxhlet methods, respectively. Regarding the a* value, both methods led to positive values, 0.03 ± 0.00^a^ and 0.13 ± 0.00^b^, respectively, indicating a certain degree of a reddish color, which was found higher (*p* < 0.05) in the oil corresponding to the Soxhlet procedure. The b* parameter of both kinds of oils showed positive values, 0.15 ± 0.00^a^ and 0.04 ± 0.00^b^, respectively, indicating a certain degree of yellowish color; nevertheless, the oil obtained by the optimized SFE-CO_2_ method showed a higher b* value (*p* < 0.05) than its counterpart from the conventional procedure.

The chroma (C*_ab_) and hue angle (h_ab_) values of the maqui oil obtained by the optimized SFE-CO_2_ method were 0.15 ± 0.00 and 78.69 ± 0.00, respectively. Those values corresponding to the Soxhlet procedure were 0.14 ± 0.00 and 17.10 ± 0.00, respectively. A positive and low chroma (C*_ab_) value indicates a slightly saturated (bright) color, more noticeable in the oil from the optimized SFE-CO_2_ method (*p* < 0.05). The hue angle value, h_ab_, was significantly (*p* < 0.05) higher in the oil corresponding to the optimized SFE-CO_2_ procedure (78.69 °); this indicates a tendency towards yellow color, while in the oil extract obtained by the Soxhlet method (17.10 °), a tendency towards red color is inferred.

**Figure 5:**
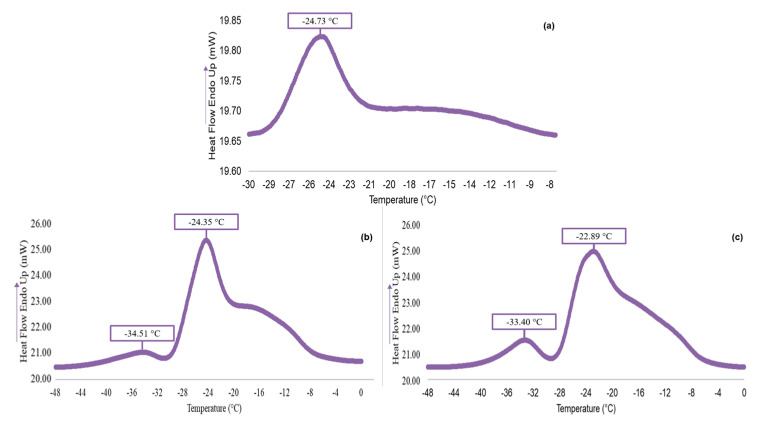
Average melting thermograms (*n* = 2) of (**a**) the freeze-dried maqui and of the maqui oil obtained by (**b**) the Soxhlet method and (**c**) the optimized SFE-CO_2_ method. ENDO UP indicates that endothermic peaks are upward.

In agreement with thermograms shown in Figure 5, it can be observed that in the freeze-dried maqui (Figure 5a), a melting peak is displayed, while in oils obtained by the Soxhlet (Figure 5b) and the optimized SFE-CO_2_ (Figure 5c) methods, two characteristic melting peaks were obtained.

The value of each peak is identified in Table 4, where the freeze-dried maqui exhibits a single melting peak at −24.73 °C, associated with the lipid content, and shows no significant differences (*p* > 0.05) compared to the peak of its corresponding oil obtained by the Soxhlet method. However, it contains a lower concentration, ΔH value of 4.09 J·g^−1^, indicating a lower lipid content due to its lower latent energy. For the oil from the freeze-dried maqui, two points corresponding to triacylglycerols with low (point 1) and high (point 2) melting points are identified, with values of −34.51 and −24.35 °C for the Soxhlet method, respectively, and −33.40 and −22.89 °C for the optimized SFE-CO_2_ method, respectively. In both methods, the onset of melting is close to −48 °C, and the end is at 0 °C. This result allows an understanding of the behavior of the oil under thermal changes and identifies the temperature conditions under which it can be stored to maintain its liquid state and preserve the appearance (both visual and tactile) of its surface. As for the freeze-dried maqui, the glass transition temperature is 36.12 ± 1.81 °C.

**Figure 6:**
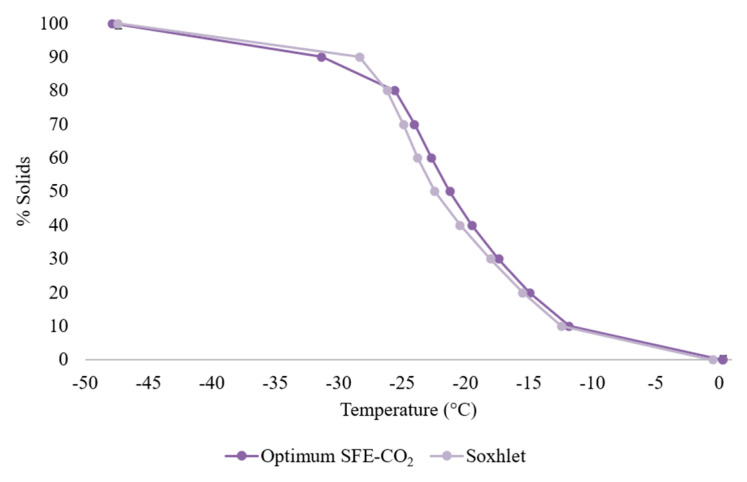
Variation in the solid content (%) as a function of temperature (°C) in the maqui oil obtained by the optimized SFE-CO_2_ and Soxhlet procedures.

In Figure 6, the solid fat content is plotted against the temperature for the oil obtained by the two extraction methods. A similar behavior is observed between the curves, leading to the conclusion that the triacylglycerol composition of both oils is similar. It is also observed that at 0 °C, the oil from the freeze-dried maqui melts completely, while the oil extracted by the Soxhlet method melted at −0.45 °C, presenting no significant differences between them (*p* > 0.05).

Concerning the chemical analysis of the oils, the results are presented in Table 5. It can be observed that both extraction methods did not lead to significant differences (*p* > 0.05) in the acidity, peroxide, *p*-anisidine, and Totox values. Regarding conjugated dienes and trienes values, significant differences (*p* < 0.05) are observed between the samples corresponding to both methods; thus, the optimized SFE-CO_2_ method led to lower (*p* < 0.05) values compared to the Soxhlet method.

The results obtained for the antioxidant capacity are shown in Table 6, where it can be observed that the oil from the freeze-dried maqui obtained by the optimum SFE-CO_2_ method had a significantly higher (ca. three times) antioxidant capacity (*p* < 0.05) than its counterpart corresponding to the Soxhlet method. The content of total phenols in the oils was calculated using the calibration curve equation with gallic acid (Table 6). In agreement with the assessment of the antioxidant behavior, the oil from the freeze-dried maqui obtained by the optimized SFE-CO_2_ method showed a significantly higher amount (*p* < 0.05) than its counterpart obtained with the Soxhlet method.

The FA analysis of the oil from the freeze-dried maqui revealed the presence of nine different FAs (Table 7). Notably, the highest amount corresponds to linoleic acid for polyunsaturated FAs, to oleic acid for monounsaturated FAs, and to palmitic acid for saturated FAs. No significant differences (*p* > 0.05) could be proved between the oils obtained by both extracting procedures regarding lauric, palmitoleic, and linoleic acids. Contrary, a significantly higher (*p* < 0.05) presence of myristic, palmitic, and linolenic FAs was detected in the oil extract corresponding to the optimized SFE-CO_2_ procedure.

Regarding the FA groups, both oils revealed high values of polyunsaturated FAs, constituting more than half of the total FAs. However, the oil obtained by the optimized SFE-CO_2_ method showed a significantly lower (*p* < 0.05) amount of monounsaturated FAs and a higher (*p* < 0.05) content of saturated FAs.

According to Table 8, the same tocopherol compounds were identified in both oil extracts. It is observed that the optimized SFE-CO_2_ method exhibits a significantly higher concentration (*p* < 0.05) compared to the Soxhlet method for all the tocopherol compounds. However, a characteristic peak corresponding to plastochromanol-8 was identified and found in higher concentrations in the extract corresponding to the Soxhlet method.

Regarding validation, the tocopherol concentration obtained by the optimized SFE-CO_2_ method exceeded the predictions from optimization. Thus, the tocopherol concentrations described in Table 8 were higher than those predicted, with the concentrations (mg·kg^−1^ oil) of 590 for α-tocopherol, 50 for α-tocotrienol, 71 for γ-tocopherol, and 3 for δ-tocopherol. Contrary, a similar value (i.e., 6 mg·kg^−1^ oil) was obtained for the β-tocopherol compound.

## 4. Discussion

Based on the experiments carried out, a yield enhancement was observed by increasing the supercritical pressure, regardless of the extracting time and the temperature. This result can be explained on the basis of the increase in CO_2_ density with the rise in pressure and an increase in the oil solubility in the solvent. The same effect of supercritical pressure on the extraction yield of olive oil was observed by Durante et al. [27] using supercritical fluid. Regarding the optimal SFE-CO_2_ performance, a ca. 7.65% oil yield was reached; this value is slightly lower than the yield obtained by the conventional procedure (ca. 8.53%).

Another response variable determined was the quantification of tocopherols (mg·kg^−1^ oil), including α-, β-, γ-, and δ-tocopherol, and α-tocotrienol. According to the results obtained, it can be affirmed that α-tocopherol is the most abundant tocopherol in the current oil, and an increase in supercritical pressure favors an increase in its concentration. However, for α-tocopherol, α-tocotrienol, and γ-tocopherol, beyond supercritical pressures of 226, 247, and 212 bar, respectively, the slope starts to decrease. This behavior can be explained on the basis that, at pressures above 200 bar, the CO_2_ density generates high densities, and as the temperature increases, its solvent capacity decreases. This inversion in solubility dependence with temperature is also reported as a retrograde solubility behavior [28].

It has been reported that the maqui oil obtained by the Soxhlet and cold-pressed methods is brighter and has greenish tones when compared to the oil obtained by the optimized SFE-CO_2_ method [21]. However, similar values were obtained for the b* parameter as both oils exhibited yellowish tones. The chroma parameter (C*_ab_) also indicated a similar saturation degree. Regarding h_ab_, all the values were close to 90 °, indicating a tendency towards yellow and brown color.

According to the melting point of four vegetable oils evaluated by DSC, the oil obtained from the freeze-dried maqui by the optimized SFE-CO_2_ extraction was found similar to sunflower, corn, and soybean oils [29]; thus, a similar thermogram profile was obtained. The temperature of glass transition (°C) is sought in the range of 20 to 80 °C, where the freeze-dried maqui used in the current study was similar to freeze-dried whole maqui found by Gómez Mattson et al. [30].

In order to achieve an accurate assessment of oil oxidation extent, different and complementary lipid oxidation indices were checked in the current study. Lipid oxidation is a complex process where different types of molecules are produced, most of which are unstable and susceptible to decomposition, resulting in lower molecular weight compounds or reacting with other molecules [31,32]. According to MINSAL [33], oils should not contain more than 0.25% of free acidity, expressed as oleic acid content, and the maximum peroxide limit should be 2.5 meq of peroxide oxygen·kg^−1^ fat at the date of manufacture and 10 meq of peroxide oxygen·kg^−1^ fat in its shelf life, stored according to the labeling. In agreement with these regulations, the oil from the freeze-dried maqui obtained by the optimized SFE-CO_2_ method could not be marketed as edible oil since it exceeds both values. However, it ought to be taken into account that the oil from the freeze-dried maqui would be classified as crude oil since it is a raw material (unrefined and without previous bleaching nor deodorization) obtained from a plant source [34]. As there is no specific regulation for this type of oil, an alternative is to compare the present values to the analytical quality limits commonly employed in edible oil such as extra virgin olive oil. According to MINSAL [33], extra virgin olive oil is exempt from this provision with a maximum acidity level allowed of 2.0% and a maximum peroxide value permitted of 20 meq peroxide oxygen·kg^−1^ oil. According to CODEX [35], the maximum dose of active oxygen·kg^−1^ oil should not overpass the 15 meq value for cold-pressed and virgin oils. Therefore, the current oil from the freeze-dried maqui obtained by the optimized SFE-CO_2_ method would comply with both national and international standards. On the basis of being a tocopherol-enriched oil, its use may be recommended in order to enhance the oxidative stability of fatty fish products including high levels of polyunsaturated FAs [36,37].

Present results regarding the hydrolytic and oxidative quality of the oil obtained are in agreement with previous values detected by Bastías et al. [21]; in such study, maqui oil was obtained by employing the Soxhlet, the conventional chloroform/methanol, and the cold-pressed procedures, all of them leading to similar values for free acidity (ca. 1.30–2.27%) and peroxide value (3.47–9.92 meq. active oxygen·kg^−1^ oil) than in the current study. Remarkably, the highest values correspond to the Soxhlet method (70 °C heating) and the lowest corresponds to the cold-pressed one, which does not include high temperatures [21]. In the present study, both oils were extracted at 60 °C; therefore, similar peroxide values (4.84 and 4.85 meq. active oxygen·kg^−1^ lipids, respectively) were detected. If the *p*-anisidine value is close to zero, it means that the oil does not contain secondary oxidative compounds that react with *p*-anisidine. Regarding conjugated dienes and trienes values, the oil sample corresponding to the optimized SFE-CO_2_ condition showed lower values of primary oxidation development (related to conjugated dienes) and also lower formation of secondary oxidation compounds, particularly those containing a carbonyl functional group (related to conjugated trienes) [23].

The formation of conjugated dienes and trienes was found to be negligible in the oil obtained from the freeze-dried maqui using the optimized SFE-CO_2_ extraction procedure. Such results would agree with the established values for extra virgin olive oil (2.50 for K232 nm and 0.22 for K270 nm) expressed by the International Olive Council (IOC) [38]. Therefore, it can be inferred that the present SFE-CO_2_ extraction method produces high-quality maqui oil with low values for the oxidation parameters.

The antioxidant capacity and the total phenolic concentration of oils from various berries (i.e., blueberry, *Vaccinium corymbosum*; red raspberry, *Rubus ideaus*; marionberry, *Rubus hybrid*; boysenberry, *Rubus hybrid*) were evaluated in previous research [39]. When comparing these results with the oil derived from optimized SFE-CO_2_ maqui extraction, a similarity was observed in the H-ORAC_FL_ antioxidant capacity of the cold-pressed boysenberry seed oil (*Rubus hybrid*), as it showed a value of 21.1 μmol TE·g^−1^ oil. However, its total phenolic concentration was found higher than in oils from different berries, ranging from 0.09 to 1.00 mg GAE·g^−1^ oil.

The oil from the freeze-dried maqui obtained by the optimized SFE-CO_2_ method contains essential FAs, namely those not synthesized by the human body and, due to their nutritional importance, should be present in the diet. These include linoleic and linolenic acids [40]. Bastías et al. [21] analyzed the FA content of the oil obtained from freeze-dried maqui by different extraction methods. As a result, polyunsaturated FAs were shown to be the most abundant group in all the cases. Linoleic acid had the highest content, representing 45.4% to 45.8%, followed by monounsaturated FAs, where oleic acid was found between 39.7% and 40.6% yields; finally, saturated FAs, with palmitic acid having a yield range of 8.5–8.6%, was the less abundant FA group. In comparison to these results, the only difference was that vaccenic acid, a cis-monounsaturated FA found in most vegetable oils, was not identified [40].

According to the CODEX [35], cottonseed oil mostly contains α-tocopherol with 136 to 674 mg·kg^−1^ oil and γ-tocopherol with 138 to 746 mg·kg^−1^ oil, similar to the levels found in the oil from the freeze-dried maqui obtained by the optimized SFE-CO_2_ method.

## 5. Conclusions

Freeze-dried maqui was checked as a source of valuable oil and tocopherol compounds. Optimized SFE-CO_2_ extraction showed an 8% yield and tocopherol compound concentrations of 735, 53, and 97 (mg·kg^−1^ oil) for α-tocopherol, α-tocotrienol, and γ-tocopherol, respectively; additionally, traces of β- and δ-tocopherol were detected. In agreement with the experimental design developed, such values were obtained by employing the factor levels of 274 bar, 240 min, and 60 °C for the three processing variables (i.e., pressure, extracting time, and temperature, respectively). Compared to the conventional Soxhlet extraction method, a higher concentration of tocopherol compounds, a higher antioxidant capacity (H-ORAC_FL_), and a higher total phenol content (22 μmol TE·g^−1^ oil and 28 mg GAE·g^−1^ oil) were observed.

To the best of our knowledge, this research provides novel and valuable information regarding the presence of tocopherol compounds (i.e., lipophilic antioxidant molecules) in maqui oil. The present results open the way to the development of tocopherol-enriched oils for preventing lipid oxidation development during the technological treatment of food substrates with low-stability to rancidity development, such as fatty fish-including foods. Valuable use of such enriched oils is expected in nutraceutical and clinical fields. 

Based on an advanced mathematical design and on the employment of a green extracting method, i.e., supercritical extraction, a novel and practical strategy is proposed for the extraction from freeze-dried maqui of lipid compounds including high levels of tocopherol molecules. In addition to being non-toxic, non-flammable, and residue-free, the SFE-CO_2_ extraction procedure has also shown to be valuable for producing a high-quality oil with remarkable levels of lipophilic (i.e., tocopherols) antioxidant compounds. The current study agrees with current research focused on obtaining highly valuable extracts including nutritional, healthy, and preserving properties from natural sources by employing sustainable technologies.

## Figures and Tables

**Table 1:** Treatment combinations according to the Box/Behnken design by RSM and oil yield (%) obtained from the freeze-dried maqui.

Extraction Number	Pressure (Bar)	Time (Min)	Temperature (°C)	Oil Yield (%)
1	74	30	48	0.00
2	187	30	60	3.20
3	300	30	48	8.81
4	300	135	60	8.82
5	187	135	48	4.38
6	187	30	35	8.30
7	187	240	35	3.71
8	300	135	35	8.09
9	187	135	48	8.34
10	74	240	48	0.00
11	187	135	48	8.16
12	187	240	60	3.79
13	74	135	35	0.00
14	300	240	48	9.20
15	74	135	60	0.00

**Table 2:** Content of tocopherol compounds (mg·kg^−1^ oil) obtained in the 15 experiments of the oil extraction from the freeze-dried maqui by SFE-CO_2_ *.

No	Content (mg·kg^−1^ Oil)				
	α- Tocopherol	α- Tocotrienol	β- Tocopherol	γ- Tocopherol	δ- Tocopherol
1	nd	nd	nd	nd	nd
2	598 ± 7 ^ab^	33 ± 3 ^ab^	Traces	93 ± 7 ^a^	Traces
3	368 ± 3 ^cde^	37 ± 1 ^a^	4.95 ± 0 ^a^	36 ± 1 ^b^	Traces
4	349 ± 2 ^cd^	29 ± 0 ^bc^	5.70 ± 0 ^ab^	33 ± 1 ^b^	6 ± 0
5	324 ± 11 ^d^	15 ± 0 ^d^	Traces	61 ± 4 ^c^	Traces
6	363 ± 4 ^cd^	26 ± 0 ^c^	Traces	44 ± 1 ^bd^	Traces
7	561 ± 12 ^a^	30 ± 2 ^bc^	Traces	78 ± 4 ^e^	Traces
8	325 ± 1 ^d^	34 ± 1 ^ab^	Traces	45 ± 0 ^bd^	Traces
9	378 ± 2 ^ce^	39 ± 0 ^a^	Traces	50 ± 0 ^cd^	Traces
10	nd	nd	nd	nd	nd
11	374 ± 4 ^ce^	47 ± 1 ^e^	6.19 ± 0 ^ab^	38 ± 0 ^bd^	Traces
12	622 ± 14 ^b^	64 ± 2 ^f^	Traces	105 ± 3 ^a^	Traces
13	nd	nd	nd	nd	nd
14	411 ± 21 ^e^	49 ± 2 ^e^	6.56 ± 1 ^b^	51 ± 3 ^cd^	Traces
15	nd	nd	nd	nd	nd

* values are mean ± SD (standard deviation) (*n* = 2). In each column, values with different superscript letters (^a–f^) indicate significant differences (*p* < 0.05); nd: not determined. Traces: lower content than 5 mg·kg^−1^ oil.

**Table 3:** Regression coefficients and *p* values of the predictive primer (Y_1_) and second (Y_2–6_) order polynomial model for the different response variables, i.e., oil yield (%) and tocopherol concentration (mg·kg^−1^ oil) obtained from the freeze-dried maqui by the SFE-CO_2_ extraction *.

Process Variables **	Responses Variables											
	*Y*_1_ (Oil Yield)		*Y*_2_ (α-Tocopherol)		*Y*_3_ (α-Tocotrienol)		*Y*_4_ (β-Tocopherol)		*Y*_5_ (γ-Tocopherol)		*Y*_6_ (δ-Tocopherol)	
	Coefficient	*p* Value	Coefficient	*p* Value	Coefficient	*p* Value	Coefficient	*p* Value	Coefficient	*p* Value	Coefficient	*p* Value
Constant	0.31		416.92		−67.62		−6.75		−134.61		4.01	
Linear												
A	0.04	0.00	9.38	0.00	0.68	0.00	0.06	0.00	1.55	0.01	−0.03	0.02
B	−0.04	0.45	−43.72	0.08	0.36	0.29	0.05	0.17	0.63	0.24	−0.10	0.06
C	0.00	0.51	−2.17	0.13	0.06	0.16	0.00	0.23	0.07	0.26	0.00	0.69
Quadratic												
A × A	-	-	−0.02	0.00	0.00	0.01	0.00	0.05	0.00	0.00	-	-
B × B	-	-	0.49	0.03	-	-	-	-	-	-	-	-
C × C	-	-	0.01	0.01	-	-	-	-	-	-	-	-
Interaction												
A × B	-	-	-	-	-	-	-	-	-	-	0.00	0.03
A × C	-	-	-	-	-	-	-	-	-	-	-	-
B × C	-	-	-	-	-	-	-	-	-	-	-	-
R^2^	80.06		96.23		77.51		83.32		79.52		65.48	
Adjusted R^2^	74.63		93.40		68.51		76.64		71.33		51.67	
SE	1.88		55.54		11.26		1.11		17.91		0.94	
MAE	1.23		33.23		7.12		0.71		12.18		0.61	
DW value	2.30	0.69	1.69	0.26	1.42	0.08	2.58	0.88	1.92	0.37	2.39	0.73
Lag 1 RA	−0.167		0.133			0.268	−0.314		0.024		−0.218	

* Abbreviations: R^2^ (regression coefficient), SE (standard error), MAE (mean absolute error), DW (Durbin–Watson), and RA (residual autocorrelation). ** Process variables: A (pressure, bar), B (temperature, °C), and C (time, min). Responses variables: (Y_1_) yield and (Y_2–6_) tocopherol concentration.

**Table 4:** Temperatures of peaks (T_Peak_), onset temperature (T_Onset_), endset temperature (T_Endset_), and ΔH (J·g^−1^) from the melting curves of different kinds of samples *.

Sample	ΔH (J·g^−1^)	T_Onset_ (°C)	T_Peak1_ (°C)	T_Peak2_ (°C)	T_Endset_ (°C)
Freeze-dried maqui	4.09 ± 1.14 ^a^	−29.98 ± 0.23 ^a^	-	−24.73 ± 0.24 ^a^	−7.14 ± 0.85 ^a^
Oil maqui by Soxhlet	81.01 ± 1.03 ^b^	−47.33 ± 1.53 ^b^	−34.51 ± 0.17 ^a^	−24.35 ± 0.08 ^a^	−0.45 ± 0.29 ^b^
Oil maqui by optimized SFE-CO_2_	78.19 ± 3.13 ^b^	−47.78 ± 0.09 ^b^	−33.40 ± 0.12 ^b^	−22.89 ± 0.04 ^b^	0.39 ± 1.17 ^b^

* values are mean ± standard deviation (SD) (*n* = 2). In each column, values with different superscript letters (^a,b^) indicate significant differences (*p* < 0.05).

**Table 5:** Chemical analyses of the maqui oil extract by the optimized SFE-CO_2_ and the conventional procedures *.

Method	Free Acidity (% Oleic Acid)	Peroxide Value (meq of Active Oxygen·kg^−1^ Oil)	*p*- Anisidine Value	TOTOX Value	Conjugated Dienes	Conjugated Trienes
Soxhlet	1.27 ± 0.06 ^a^	4.84 ± 0.25 ^a^	0 ^a^	9.69 ± 0.50 ^a^	0.053 ± 0.010 ^a^	0.014 ± 0.001 ^a^
Optimized SFE-CO_2_	1.20 ± 0.13 ^a^	4.85 ± 0.35 ^a^	0 ^a^	9.69 ± 0.69 ^a^	0.005 ± 0.000 ^b^	0.000 ± 0.000 ^b^

* values are mean ± standard deviation (SD) (*n* = 2). In each column, values with different superscript letters (^a,b^) indicate significant differences (*p* < 0.05).

**Table 6:** H-ORAC_FL_ value and total phenol content of the oil obtained from the freeze-dried maqui by the optimized SFE-CO_2_ and the conventional procedures *.

Method	H-ORAC_FL_ (μmol TE·g^−1^ Oil)	Total Phenols (mg GAE·g^−1^ Oil)
Soxhlet	7.01 ± 0.09 ^a^	14.69 ± 0.08 ^a^
Optimized SFE-CO_2_	21.91 ± 0.03 ^b^	28.44 ± 0.23 ^b^

* values are mean ± standard deviation (SD) (*n* = 2). In each column, values with different superscript letters (^a,b^) indicate significant differences (*p* < 0.05).

**Table 7:** Fatty acid (FA) (g·100 g^−1^ total FAs) profile of the oil obtained from the freeze-dried maqui by the optimized SFE-CO_2_ and the conventional procedures *.

Systematic Name	Abbreviation Name	Soxhlet Method	Optimized SFE-CO_2_ Method
Lauric acid	C12:0	0.17 ± 0.01 ^a^	0.22 ± 0.02 ^a^
Myristic acid	C14:0	0.58 ± 0.01 ^a^	0.65 ± 0.00 ^b^
Palmitic acid	C16:0	8.25 ± 0.01 ^a^	8.70 ± 0.02 ^b^
Palmitoleic acid	C16:1 9c	0.24 ± 0.01 ^a^	0.26 ± 0.00 ^a^
Stearic acid	C18:0	2.47 ± 0.00 ^a^	2.36 ± 0.01 ^b^
Oleic acid	C18:1 9c	33.54 ± 0.01 ^a^	32.99 ± 0.04 ^b^
Cis-Vaccenic acid	C18:1 7c	2.71 ± 0.00 ^a^	2.66 ± 0.01 ^b^
Linoleic acid	C18:2 9c 12c	50.45 ± 0.02 ^a^	50.41 ± 0.05 ^a^
Linolenic acid	C18:3 9c 12c 15c	1.59 ± 0.00 ^a^	1.75 ± 0.01 ^b^
Total saturated fatty acids		11.47 ^a^	11.92 ^b^
Total monounsaturated fatty acids		36.50 ^a^	35.92 ^b^
Total polyunsaturated fatty acids		52.04 ^a^	52.16 ^a^

* values are mean ± standard deviation (SD) (*n* = 2). In each row, values with different superscript letters (^a,b^) indicate significant differences (*p* < 0.05).

**Table 8:** Tocopherol compounds content (mg·kg^−1^ of oil) of the oil obtained from the freeze-dried maqui by the optimized SFE-CO_2_ and the conventional procedures *.

Method	Concentration (mg·kg^−1^ Oil)					
	α- Tocopherol	α- Tocotrienol	PC-8	β- Tocopherol	γ- Tocopherol	δ- Tocopherol
Soxhlet	347 ± 2 ^a^	28 ± 0 ^a^	15 ± 1 ^a^	Traces	50 ± 1 ^a^	Traces
Optimized SFE-CO_2_	735 ± 19 ^b^	53 ± 0 ^b^	10 ± 0 ^b^	5 ± 1	97 ± 4 ^b^	7 ± 1

* values are mean ± SD (*n* = 2). In each column, values with different letters as superscripts (^a,b^) indicate significant differences (*p* < 0.05). Traces: lower levels than 5 mg·kg^−1^ oil. PC-8: Plastochromanol-8.

## Data Availability

All the data are contained within the manuscript.

## Supplementary Materials

The following supporting information can be downloaded at: https://www.mdpi.com/article/10.3390/antiox13070845/s1, Table S1: Goodness-of-fit test of the statistical analysis carried out in the study.
