# Supplementary material for: Optimization of Oil and Tocopherol Extraction from Maqui (Aristotelia chilensis (Mol.) Stuntz) by Supercritical CO2 Procedure
Source: Antioxidants (Basel). 2024 Jul 15;13(7):845. doi: 10.3390/antiox13070845 (PMC11273519; doi:10.3390/antiox13070845)
Supplement: Supplementary file 1 [file antioxidants-13-00845-s001.zip › antioxidants-3072261-supplementary.pdf]

Supplementary Material: TABLE S1

Goodness-of-fit test of the statistical analysis carried out in the study

Analysis	R^2	*adjusted* R^2	*CV*	*P-value*	*Interpretations*
α- tocopherol (mg·kg^{-1} oil)	*1.00*	*0.99*	*3.56*	*0.0025*	*The model explains almost all the variability with excellent fit (high adjusted R^2) and is statistically significant.*
α- tocotrienol (mg·kg^{-1} oil)	*1.00*	*1.00*	*0.64*	*0.0001*	*The model is perfect in explaining the variability, with very low variability and is highly significant.*
Plastocromanol-8 (mg·kg^{-1} oil)	*0.98*	*0.97*	*4.04*	*0.0098*	*The model explains most of the variability with good accuracy and is significant.*
β- tocopherol (mg·kg^{-1} oil)	*0,84*	*0.77*	*16.36*	*0.0811*	*The model explains a reasonable amount of variability but is not statistically significant.*
γ- tocopherol (mg·kg^{-1} oil)	*0.99*	*0.98*	*5.35*	*0.0069*	*The model explains very well the variability and is significant.*
δ- tocopherol (mg·kg^{-1} oil)	*0.87*	*0.80*	*21.72*	*0.0678*	*The model has a good fit but is not statistically significant.*
Oil yield (%)	*0.27*	*0.23*	*9.36*	*0.0184*	*The model does not explain correctly the variability but is significant.*
ΔH (J·g^{-1})	*1.00*	*0.99*	*5.23*	*0.0002*	*The model is perfect in explaining the variability and is highly significant.*
TOnset (°C)	*0.99*	*0.98*	*3.03*	*0.0012*	*The model explains almost all the variability and is significant.*
TPeak1 (°C)	*0.93*	*0.90*	*0.61*	*0.0334*	*The model has a good fit and is significant.*
TPeak2 (°C)	*0.94*	*0.94*	*0.88*	*0.0063*	*The model explains*

					correctly the variability and is significant.
TEndset (°C)	*0.94*	*0.90*	*50.18*	*0.0147*	*The model explains correctly the variability, but with greater relative variability, and is significant.*
*L**	*1.00*	*1.00*	*8,1E-8*	*<0.0001*	*The model is perfect and highly significant.*
*a**	*1.00*	*1.00*	*7.5E-7*	*<0.0001*	*The model is perfect and highly significant.*
*b**	*1.00*	*1.00*	*1.2E-6*	*<0.0001*	*The model is perfect and highly significant.*
*C*ab*	*1.00*	*1.00*	*2.6 E-8*	*<0.0001*	*The model is perfect and highly significant.*
Hab	*1.00*	*1.00*	*6.2 E-7*	*<0.0001*	*The model is perfect and highly significant.*
Free acidity (% oleic acid)	*0.11*	*0.00*	*11.26*	*0.6701*	*The model does not explain correctly the variability and is not significant.*
Peroxide value (meq of active oxygen·kg^{-1} oil)	*2.7E-6*	*0.00*	*8.82*	*0.9983*	*The model does not explain any variability and is not significant.*
p- anisidine value	-	-	-	-	-
TOTOX value	*6.2 E-6*	*0.00*	*8.83*	*0.9975*	*The model does not explain any variability and is not significant.*
Conjugated dienes	*0.92*	*0.88*	*34.48*	*0.0408*	*The model explains correctly the variability and is significant.*
Conjugated trienes	*1.00*	*1.00*	*7.41*	*0.0014*	*The model is perfect and highly significant.*
H-ORACFL (µmol TE·g^{-1} oil)	*1.00*	*1.00*	*0.64*	*<0.0001*	*The model is perfect and highly significant.*
Total phenols (mg GAE·g^{-1} oil)	*1.00*	*1.00*	*1.10*	*0.0003*	*The model is perfect and highly significant.*
Lauric acid (g·100 g^{-1} total FAs)	*0.74*	*0.61*	*11.04*	*0.1408*	*The model explains a reasonable amount of the variability in lauric acid but is not significant.*
Myristic acid (g·100 g^{-1} total FAs)	*0.98*	*0.97*	*1.06*	*0.0103*	*The model explains correctly the variability and is significant.*

Palmitic acid ($g \cdot 100\ g^{-1}$ total FAs)	*0.99*	*0.99*	*0.31*	*0.0035*	*The model explains very well the variability and is significant.*
Palmitoleic acid ($g \cdot 100\ g^{-1}$ total FAs)	*0.86*	*0.79*	*2.46*	*0.0737*	*The model has a good fit but is not statistically significant.*
Stearic acid ($g \cdot 100\ g^{-1}$ total FAs)	*0.99*	*0.98*	*0.37*	*0.0065*	*The model explains well the variability and is significant.*
Oleic acid ($g \cdot 100\ g^{-1}$ total FAs)	*0.99*	*0.99*	*0.11*	*0.0046*	*The model explains very well the variability and is significant.*
Cis-Vaccenic acid ($g \cdot 100\ g^{-1}$ total FAs)	*0.92*	*0.87*	*0.37*	*0.0428*	*The model explains correctly the variability and is significant.*
Linoleic acid ($g \cdot 100\ g^{-1}$ total FAs)	*0.20*	*0.00*	*0.12*	*0.5485*	*The model does not explain correctly the variability and is not significant.*
Linolenic acid ($g \cdot 100\ g^{-1}$ total FAs)	*1.00*	*1.00*	*0.38*	*0.0016*	*The model is perfect and highly significant.*
Total saturated fatty acids (TSFA) ($g \cdot 100\ g^{-1}$ total FAs)	*0.63*	*0.45*	*0.12*	*0.2037*	*The model explains a reasonable amount of variability but is not significant.*
Total monounsaturated fatty acids (TMUFA) ($g \cdot 100\ g^{-1}$ total FAs)	*0.99*	*0.98*	*0.13*	*0.0066*	*The model explains very well the variability and is significant.*
Total polyunsaturated fatty acids (TPUFA) ($g \cdot 100\ g^{-1}$ total FAs)	*0.99*	*0.98*	*0.30*	*0.0059*	*The model explains very well the variability and is significant.*

In summary, most models explain correctly the variability of the dependent variables and many of them are statistically significant. Variables related to L^*, a^*, b^*, C^*_{ab}, and H_{ab} show perfect models with high significance, whereas variables such as free acidity, peroxide value, and TOTOX value are not correctly explained by the models and are not significant.
